# Traditional acupuncture and laser acupuncture in chronic nonspecific neck pain: study protocol for a randomized controlled trial

**DOI:** 10.1186/s13063-022-06349-y

**Published:** 2022-05-16

**Authors:** Rafaela Peron, Érika Patrícia Rampazo, Richard Eloin Liebano

**Affiliations:** 1 Department of Physical Therapy, Federal University of São Carlos (UFSCar), Rodovia Washington Luis, km 235, São Carlos, SP CEP 13565-905 Brazil; 2 Physiotherapeutics Resources Laboratory, Department of Physical Therapy, Federal University of São Carlos (UFSCar), São Carlos, Brazil

**Keywords:** Neck pain, Traditional acupuncture, Laser acupuncture, Study protocol, Randomized controlled trial

## Abstract

**Background:**

Nonspecific neck pain is a multifactorial and very common condition in adult individuals, traditional acupuncture (TA) and laser acupuncture (LA) may be treatment options for certain individuals in such a condition. However, no reports were found in the literature comparing the effectiveness of TA and LA in cases of chronic nonspecific neck pain. Therefore, the aim of the present study is to investigate the effectiveness of TA and LA therapies in individuals with chronic nonspecific neck pain, noting which one is more efficient for this condition. The result of this research will have direct implications for pain management and, consequently, may benefit individuals suffering from nonspecific chronic neck pain.

**Methods/design:**

This will be a controlled and randomized clinical trial. Eighty-four individuals will be recruited and distributed equally and randomly into 3 groups: TA (which will receive the acupuncture treatment with needles), LA (which will receive the laser acupuncture treatment), and Sham (who will receive the placebo intervention). The acupuncture points (*Tianzhu*, *Fengchi*, *Jianjing*, and *Jianzhongshu*) will be stimulated bilaterally. The primary outcome will be pain intensity, determined using the Numerical Rating Scale. The secondary outcomes will be pressure pain threshold, temporal summation of pain, conditioned pain modulation, use of analgesic medicines after treatment, and the global perceived effect scale. The assessments will be performed immediately before and after the treatment, which will be a single session, at the follow-up and 1 month after the end of the treatments; evaluation will be made of the pain intensity and the global perceived effect. Statistical analysis of the data obtained will consider a significance level of *p* < 0.05.

**Discussion:**

This study will provide evidence concerning the effects of LA treatment, in comparison with TA and sham intervention, leading to benefits for individuals suffering from chronic nonspecific neck pain.

**Trial registration:**

Brazilian Registry of Clinical Trials - ReBEC RBR-7vbw5gd. Date of registration: August 06th, 2021.

## Administrative information

*Note: The numbers in curly brackets in this protocol refer to SPIRIT checklist item numbers. The order of the items has been modified to group similar items.

**Table:** 

Title {1}	Traditional acupuncture and laser acupuncture in chronic nonspecific neck pain: study protocol for a randomized controlled trial
Trial registration {2a and 2b}	RBR-7vbw5gd (Brazilian Registry of Clinical Trials - ReBEC)
Protocol version {3}	version 2
Funding {4}	This work will be financed in part by the Coordenação de Aperfeiçoamento de Pessoal de Nível Superior -, Brazil (CAPES – Finance code 001).
Author details {5a}	Rafaela Peron^a^, Érika Patrícia Rampazo^b^, Richard Eloin Liebano^c*^ ^a^Department of Physical Therapy, Federal University of São Carlos (UFSCar), Rodovia Washington Luis, km 235, São Carlos, São Paulo, Brazil, CEP 13565-905. E-mail: rafaelaperoncardoso@gmail.com ^b^Department of Physical Therapy, Federal University of São Carlos (UFSCar), Rodovia Washington Luis, km 235, São Carlos, São Paulo, Brazil, CEP 13565-905. E-mail: erika.rampazo@gmail.com ^c^Department of Physical Therapy, Federal University of São Carlos (UFSCar), Rodovia Washington Luis, km 235, São Carlos, São Paulo, Brazil, CEP 13565-905. E-mail: liebano@gmail.com. *Author for correspondence. Tel.: +55 16 3351 8985. Physiotherapeutics Resources Laboratory, Department of Physical Therapy, Federal University of São Carlos (UFSCar).
Name and contact information for the trial sponsor {5b}	None
Role of sponsor {5c}	Not applicable.

## Introduction

### Background and rationale {6a}

Neck pain is a very common condition that causes considerable disability and economic losses [1, 2]. Between the years 1990 and 2017, high indices of neck pain were reported in 195 countries, so it can be considered a serious global public health problem, ranked fourth among the causes of disability, with an annual prevalence rate higher than 30% [2, 3]. It is more common in women and usually increases with age, reaching peaks in the age groups 45–49 and 50–54 years for men and women, respectively, with a decrease for older persons [2]. Several risk factors are associated with neck pain, including obesity and a sedentary lifestyle. It results in restrictions and disabilities that can have major impacts on the physical, social, and psychological well-being of the individual [2, 4, 5]. Neck pain can be classified according to its etiology. It is considered specific when there are anatomical changes resulting from dysfunctions in neck structures, such as the deterioration of nerve roots, capsule damage, nerve root compression, or fractures [3]. Nonspecific neck pain is multifactorial in nature and is not associated with a specific disease or anatomical modification [6, 7]. Neck pain is classified according to the duration of symptoms, being acute (up to 1 month), subacute (1 to 3 months), and chronic (at least 3 months) [8–10]. Chronic neck pain is defined as any continuous pain in the cervical spine region between the base of the head and the upper shoulder, lasting at least 12 weeks and usually accompanied by reduced cervical movement [11].

Various strategies can be used for the treatment of neck pain, including acupuncture, which originated over 3000 years ago and constitutes part of traditional Chinese medicine [12–15]. The technique, performed with needles, produces good results and is effective in reducing pain and disability [16–23]. It has been shown to provide clinical benefits in individuals with neck pain, so it is a viable option for use by health services [24]. It is considered a safe treatment, with serious side effects being rare, compared to conventional techniques used for the treatment of pain [25, 26].

There are now alternative acupuncture techniques available that do not use needles, such as laser acupuncture (LA), which combines the use of the traditional Chinese acupuncture points with stimulation by laser light energy [15, 27, 28]. The lasers most indicated for LA are red or infrared, due to their deeper penetration into human tissue, with powers in the range from 5 to 500 mW (Low-Level Laser Therapy (LLLT)) [28–30]. Laser acupuncture acts to enhance enzymatic defense systems, increasing plasma levels of superoxide dismutase (SOD), glutathione reductase (GRd), reduced glutathione (GSH), and catalase. It assists in maintaining the integrity of the redox cycle and can promote reductions of malondialdehyde (MDA, a plasma marker of oxidative stress), the inflammation markers C-reactive protein (CRP) and interleukin-6 (IL-6), and glutathione peroxidase (GPx). It can reduce oxidative stress damage, inflammation, joint edema, and pain in patients with rheumatoid arthritis [31].

An obvious difference between acupuncture using needles and LA is that the laser enables the stimulation of acupuncture points without damaging the skin tissue [30]. The laser induces a photochemical reaction in the cells, in a process known as photobiomodulation, at acupuncture points selected using the same rules as traditional Chinese acupuncture [32, 33]. Using an optical power of around 40 mW, the effect of LA is similar to that obtained with metal needles [28], so it can be an attractive option for the treatment of chronic pain [34].

Acupuncture using needles is a widely accepted technique that provides good results and can be effective in reducing chronic neck pain [16, 17, 19, 20, 35–44].

Meanwhile, the clinical use of LA is increasing, since it offers the advantages of being totally painless, non-invasive, and safe [30, 33, 43], and it can be used with people who have a phobia of needles. There are few studies in the literature using LA for cervical and pain conditions, such as chronic cervical syndrome, myofascial pain syndrome, spondylosis, temporomandibular disorders, and musculoskeletal pain, with positive results, strengthening the use of laser acupuncture [39, 45–48].

However, more studies are needed, and until the present moment, no studies were found that compared traditional acupuncture with LA in individuals with chronic nonspecific neck pain. There is a need for thorough research concerning LA, with robust methodologies, large sample sizes, and standardized and reproducible parameters, in order to increase understanding of this therapy and establish possible future clinical applications [30, 49, 50].

### Objectives {7}

The aim of this study is to compare the effects of traditional acupuncture and laser acupuncture therapies in individuals with chronic nonspecific neck pain.

#### The primary objective

The primary objective is to assess the pain intensity at rest and during cervical active movements (flexion, extension, left and right lateral bending, and left and right rotation) using the Numerical Rating Scale (NRS), immediately after the end of the therapies and with follow-up after 1 month.

#### The secondary objective

The secondary objective is to evaluate the pain using pressure pain threshold (PPT); temporal summation (TS) of pain; conditioned pain modulation (CPM); global perceived effect (GPE), immediately after the end of the therapies; and consumption of analgesic medications for neck pain, and global perceived effect (GPE) will follow up after 1 month.

### Trial design {8}

This study will be a randomized controlled clinical trial (RCT) with a blinded assessor, conducted according to the established standards for reporting clinical trials: Consolidated Standards of Reporting Trials (CONSORT) and Standards for Reporting Interventions in Clinical Trials of Acupuncture (STRICTA). The acupuncture style adopted will be traditional acupuncture and laser acupuncture, and the protocol was developed following the recommendations of Standard Protocol Items: Recommendations for Interventional Trials (SPIRIT).

## Methods: participants, interventions, and outcomes

### Study setting {9}

The study will be undertaken at the Physiotherapy Department of the Federal University of São Carlos (UFSCar), in the city of São Carlos, São Paulo State, Brazil.

### Eligibility criteria {10}

After screening using an online form, the potential participants will be registered, and checks will be performed considering the inclusion and exclusion criteria.

### Inclusion criteria

Individuals will be recruited if they meet the following conditions: age between 18 and 60 years, complete primary education of both sexes, neck pain complaints (pain and stiffness, for a minimum period of 3 months, reported during screening), score ≥ 3 for pain intensity on the Numerical Rating Scale (NRS), consent to random allocation and present consent and in accordance with the conditions of the treatments, and possible adverse effects, presented and explained in the consent form.

### Exclusion criteria

Individuals will be excluded if they present fracture or history of vertebral injury, spondylolisthesis, spinal canal stenosis, ankylosing spondylitis, signs of radiculopathy, cancer, acute infections, hemorrhagic diseases, rheumatologic diseases, systemic inflammatory diseases, diagnostic changes in skin sensitivity, hearing deficits (uncorrected), difficulty in answering questionnaires, in conditions of pregnancy, puerperium and breastfeeding; individuals who have started or underwent any type of intervention or treatment in the last 3 months; individuals who will be orientated not to ingest any sedative, anti-inflammatory and analgesic medications (narcotics and non-narcotics), and any type of alcoholic substance in the last 48 h before the evaluation; individuals who do not comply with the conditions and treatments, or who refuse to participate in the research, will be excluded; individuals who could not remain in the ventral decubitus position, upper limbs along the body and cervical in neutral position during the time of interventions; and individuals will be free to discontinue the treatment at any time, and if they make the most recent data, they will be computed for analysis, as well as the reason for the interruption.

### Who will take informed consent? {26a}

Data collection will only start after the participants have signed an informed consent form, which will be explained by the evaluator.

### Additional consent provisions for collection and use of participant data and biological specimens {26b}

Not applicable for this study.

### Interventions

#### Explanation for the choice of comparators {6b}

No studies were found in the literature that effectively compared traditional acupuncture (TA) with laser acupuncture (LA) for the treatment of chronic nonspecific neck pain. Therefore, the proposed research will focus on evaluating and comparing the changes in pain symptoms and neck functional capacity, after the application of TA and LA in cases of chronic nonspecific neck pain.

#### Intervention description {11a}

**Fig. 1:**
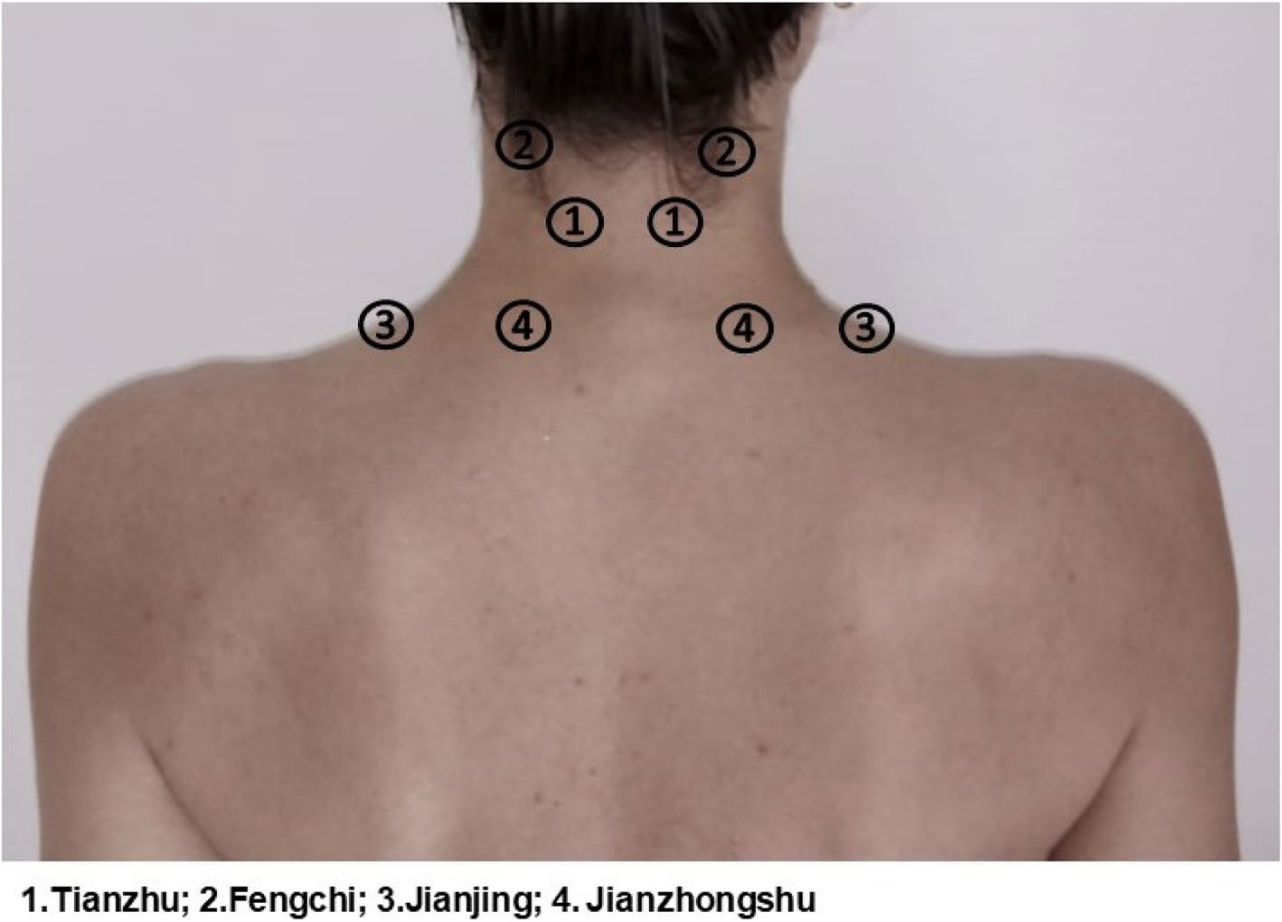
Acupuncture points that will be used in therapies

The interventions will follow a standardized therapy protocol, and the assessments will be performed immediately before and after the single treatment session. Four acupuncture points (*Tianzhu*, *Fengchi*, *Jianjing*, and *Jianzhongshu*) will be stimulated bilaterally with needles, laser, or sham. The participant will be positioned in a ventral decubitus position, upper limbs along the body, and cervical in neutral position and asked to rest for 5 min, prior to the intervention. At the locations where the therapies will be applied, the skin will be hygienized with gauze and 70% alcohol solution. A dermatograph pencil (COLOURED®) will be used to draw circles with diameters of 2 cm around the application points. The same researcher, an acupuncturist with specialization in the field and 9 years of clinical experience, will perform all the interventions (R3). The individuals will be asked not to use any other treatment for neck pain during the study period. All the data collected about the participants will be confidential (Fig. 1).

##### Traditional acupuncture (TA)

For the TA group, the interventions will be performed using disposable Goldlife® needles (0.25 mm diameter, 40 mm length; Brazilian product registration number MS80553910002) supplied in packages containing 10 surgical steel needles and a needle guide tube. Using the guide tube, the needles will be appropriately positioned in the centers of the areas demarcated on the skin and manually inserted into the muscle to a depth of 20 mm. The acupuncture points (*Tianzhu*, *Fengchi*, *Jianjing*, and *Jianzhongshu*) will be stimulated bilaterally. The manual needle stimulation technique known as sparrow pecking will be used [35, 51, 52]. After the stimuli, the needles will be left in position for 20 min [43, 46, 53], followed by the removal using a stainless steel collecting cup and disposal in an appropriate container for sharps (Descarpack®), in each session, new needles will be used.

##### Laser acupuncture (LA)

For the LA group, the interventions will be performed using Recover® laser (MMO, São Carlos, SP, Brazil). The laser will be placed at the specific acupuncture points: Tianzhu, Fengchi, Jianjing, and Jianzhongshu. The laser tip (0.03 cm^2^ of beam area) will be positioned with the “probe” head directly on the acupuncture point, in a perpendicular position and in direct contact with the skin, and every point will be treated for 2 min, totaling 16 min of treatment, plus 4 min of rest, to standardize with the traditional acupuncture time, which is 20 min. The parameters (Table 1) are based on a previous work using LA for neck pain [46], following the criteria suggested by Baxter [50].

**Table 1:** Laser acupuncture parameters

Parameters	Infrared laser
*Center wavelength [nm]*	*808*
*Operating mode*	*Continuous wave*
*Output power [mW]*	*100*
*Power density [W/cm*^*2*^*]*	*3.33*
*Beam profile*	*unique*
*Irradiance at target [W/cm*^*2*^*]*	*3.33*
*Energy density [J/cm*^*2*^*]*	*333*
*Total time per point [s]*	*120*
*Radiant energy per point [J]*	*10*
*Number of points irradiated*	*8*
*Area irradiated [cm*^*2*^*]*	*0.03*
*Total radiant energy [J]*	*80*
*Angle of application*	*90°*
*Application technique*	*Contact*

##### Sham [S]

For the sham group intervention [S], individuals should be treated with the same care as the current intervention group, including therapy time, attention to the individual, and temperature in the treatment room. The placebo intervention will be performed as a control for groups TA and AL. For the placebo intervention, the same device as the real intervention group will be used (Recover® from MMO, São Carlos, SP, Brazil); however, the treatment will be simulated with a laser probe disabled by the manufacturer, and the device maintain the common acoustic signals during therapy [16]. Participants will not be informed of the laser probe inactivation. The criteria for selecting points, parameters, and dose are identical to those used in the AL group (Table 1), including the time of application and placement of the probe at the acupuncture points: Tianzhu, Fengchi, Jianjing, and Jianzhongshu.

#### Criteria for discontinuing or modifying allocated interventions {11b}

There will be no changes in the allocations and no migration of individuals between the groups will be allowed. If individuals discontinue treatment, recent data will be computed for the analyses according to the intention-to-treat principle, and the motive for the desistence will be recorded.

#### Strategies to improve adherence to interventions {11c}

To minimize data loss, all the participants in the trial will receive a card with the date and time of session. The therapist responsible for the session will send a WhatsApp message from a cellphone account used only for this research, confirming the session, 1 day in advance. For the evaluations and reevaluations, the blinded evaluator will contact the participants by WhatsApp, confirming the initial and final evaluations, as well as the follow-up 1 month after the end of the intervention, which will be performed by telephone. In cases of abandonment or impossibility of continuing the study, the data will be analyzed according to an intention-to-treat protocol.

#### Relevant concomitant care permitted or prohibited during the trial {11d}

Concomitant treatments such as continuous use medications for hypertension and diabetes will be permitted during the trial. The participants will not be permitted to start any new type of intervention or activity during the trial period.

#### Provisions for post-trial care {30}

Individuals will be treated in one session, and they will receive a document with all the care that must be considered. In addition, they will have the telephone numbers of the researchers and the department where the study will be carried out. They will also receive a diary of records of medications consumed after the proposed therapies. The individuals will be monitored for 1 month after the intervention and will be reassessed by phone. The therapies normally do not bring greater health risks to those who participate. However, if in any case the individual has any adverse effect, he/she will be referred to the University’s health center for specific care.

### Outcomes {12}

The primary outcome will be the pain intensity. It will be evaluated using the numerical rating scale (NRS). Secondary outcomes will be as follows: pressure pain threshold (PPT), temporal summation of pain (TS), conditioned pain modulation (CPM), use of analgesic medications after treatments, and Global Perceived Effect Scale (GPE). These outcomes will be evaluated before and after the treatments. Evaluation using the NRS and the global perceived effect scale will be performed at the end of the 1-month follow-up period.

#### Numeric Rating Scale (NRS)

The NRS employs a simple and easy-to-use scale, where the individual is asked to indicate the intensity of the current pain, using a scale ranging from 0 (“no pain”) to 10 (“worst pain imaginable”) [54]. The participants will be instructed to rate the pain experienced at rest and during movements (flexion, extension, lateral inclination to the left and right, and rotation to the left and right). All before-after data will be individually recorded and compared for their respective positions.

#### Pressure pain threshold (PPT)

Measurements will be made using a pressure algometer (Somedic®, Hӧrby, Sweden), with the tip of the instrument (1 cm^2^) positioned on cervical zygapophysial joints C5/6 [55], between C7 and the acromion, bilaterally at the midpoint of the upper trapezius muscle [56], and on the middle third of the right tibialis anterior muscle. All these points will be previously marked with a dermatograph pencil. The algometer probe will be positioned perpendicular to the skin, and pressure will be applied at a rate of ~ 40 kPa/s. Three consecutive PPT measurements will be made, with an interval of 30 s between them, and the average value will be recorded. The participants will be instructed to close their eyes and to press the algometer sensor when the pressure sensation becomes painful, with the corresponding value being recorded. A test will be performed to familiarize the individuals with the algometer, explaining the procedure, and a demonstration will be made using the thenar region of the hand. During the actual measurements, the individuals will not be allowed to see the algometer. The intra-examiner reliability of the PPT measurements will be evaluated using measurements at the same points for 10 healthy and asymptomatic individuals, performed by a single examiner at intervals of 48 h. The reliability will then be estimated by calculating intraclass correlation coefficients (ICC) [55–58].

#### Temporal summation of pain (TS)

Measurements will be made using the pressure algometer (Somedic®, Hӧrby, Sweden), applying a total of 10 stimuli, at a rate of 40 kPa/s, using the average value from the previous algometry test performed on the most painful upper trapezius muscle (or the dominant side if there is no evident difference). Each stimulus will be maintained for 1 s, using a timer to ensure the correct intervals (1 s). The individuals will be asked about their pain at the 1st, 5th, and 10th seconds of stimulus, using the NRS. In order to avoid any interference, the test will begin 5 min after the PPT [58, 59].

#### Conditioned pain modulation (CPM)

Cold nociceptive stimulus will be employed. Individuals will receive instructions before the test. Conditioned stimulus will be provided by the hand on the side ipsilateral to the region of the greatest neck pain, which will be immersed for 1 min in a water bath kept at 22 °C, in order to standardize the hand temperature. After that, the same hand will be submerged (up to the wrist) for 1 min in ice water (kept at 4 °C). After 30 s, the individual will be asked to report (using the NRS) the intensity of the pain in the hand, caused by the ice water. After immersion for 1 min, the patient will be asked to remove the hand from the ice water. The PPT will be performed (between C7 and the acromion, at the mid-point of the upper trapezius muscle), using the side contralateral to the immersed hand. The PPT evaluation will be performed before and immediately after the cold stimulus. The mean PPT value before the stimulus will be subtracted from the mean value after the stimulus, with a lower value indicating that the endogenous system is less efficient in inhibiting pain [58, 60, 61].

#### Use of analgesic medicines

It will be important to record the use of analgesic medications, in order to determine whether their use decreased after treatment. The participants will be asked to report all the medications used. This information will be collected and recorded. In tables, the analgesic medications will be converted into opioids, considering the equianalgesic dose of morphine [62] and non-opioids and considering the equianalgesic dose of acetaminophen (paracetamol) [63].

#### Global Perceived Effect Scale (GPE)

The Global Perceived Effect Scale is used to assess the perception of the individual regarding any improvement, comparing the initial symptoms with the current condition. This scale, which has been translated into Portuguese and validated, provides good reliability when applied to musculoskeletal disorders. It uses 11 points, ranging from − 5 (much worse) to 0 (no change) and to 5 (completely recovered). After treatment, the participants will be asked: “Compared to when this problem first started, how would you describe the condition of your neck today?” A higher score indicates an improved condition [64, 65].

### Participant timeline {13}

**Fig. 2:**
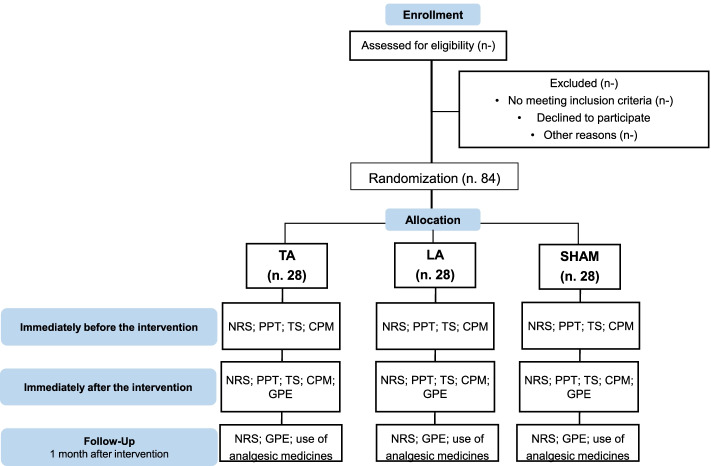
Flow diagram of the randomized clinical trial. NRS, Numeric Rating Scale; PPT, pressure pain threshold; CPM, conditioned pain modulation; GPE, global perceived effect scale

The participant timeline is presented in Fig. 2.

### Sample size {14}

The calculation of the sample size considered a difference of 1.5 points between the groups, which would be a clinically relevant value for the outcome of neck pain intensity, as measured using the Numeric Rating Scale (NRS) [64, 66], with an estimated standard deviation of 1.6 points, based on a scientific clinical study [43]. There are a statistical power of 80% and a possible sample loss of 20%, considering individuals who for any reason want to interrupt the intervention. Thus, 84 individuals will be needed in total, 28 in each group (TA, LA, and Sham). The processing of the sample calculation was performed using the Minitab software, v.17, State College, PA.

### Recruitment {15}

The dissemination and recruitment of participants for the research will be done through advertisements (internet, radio, and posters) in the city of São Carlos. The dissemination of the research will also be carried out in all health units in the city and in the university’s internal media, with the collaboration of other research departments for the recruitment of individuals.

### Assignment of interventions: allocation

#### Sequence generation {16a}

The individuals included in the study will be randomized via the website (randomization.com). In this selection process, the site will generate a random sequence, and each individual will be randomized into a single group using the permuted block method, 14 blocks with 6 each, totaling 84 in total. Then, they will have the same probability of being drawn and allocated into one of the 3 study groups: traditional acupuncture [TA] (individuals who will receive acupuncture intervention with needles), laser acupuncture [AL] (individuals who will receive laser acupuncture intervention), and sham [S] (subjects who will receive placebo intervention). The researcher, responsible for the randomization, will have no other role in this study.

#### Concealment mechanism {16b}

After the website (http://randomization.com) generates a numerical sequence for an allocation, the concealment of selected individuals will be achieved through opaque sealed envelopes, which will be stored in a safe cabinet that only the allocation investigator will have access to. The envelopes will be opened immediately before the intervention by the researcher responsible for applying the treatments.

#### Implementation {16c}

This study will have five researchers who will be independently specialized, and each one of them will have a function. The researcher, mastering (R1), will be responsible for recruiting and registering the participants. The researcher (R2) will be responsible for randomization and for generating a random allocation sequence. The researcher (R3) will only be responsible for applying the interventions (TA, LA, and SHAM). The researcher (R4) will carry out all research evaluations (NRS, PPT, TS, CPM, GPE, use of analgesic medications after treatments). The last researcher (R5) will analyze and register the data.

### Assignment of interventions: blinding

#### Who will be blinded {17a}

The evaluator will be blinded to the randomization and intervention processes, be responsible only for recruitment and evaluations, and receive no information regarding the allocation of the individuals to the groups. The participants and the researcher responsible for the treatment will not be blinded, due to the nature of the interventions.

#### Procedure for unblinding if needed {17b}

The evaluator will not be allowed to unblock the blinding. However, the acupuncturist researcher responsible for applying the treatments will not be blinded.

### Data collection and management

#### Plans for assessment and collection of outcomes {18a}

The interventions will follow an application protocol. The evaluator will be trained in the application of the questionnaires and tests, while intra-examiner reliability will be estimated by calculation of ICC.

#### Plans to promote participant retention and complete follow-up {18b}

The participants will be provided with guidance when they sign the informed consent form and commit to attending on the scheduled treatment dates. They will receive a card with the dates of the treatment sessions, a contact telephone number, and a WhatsApp contact number used only for this research. This will ensure that they receive the necessary attention and will assist in fully accompanying them during the research. If individuals abandon the trial, the reasons will be recorded, the most recent data will be computed, and analysis will be performed using the intention-to-treat principle.

#### Data management {19}

A data management plan was created using the dmptool template. All the data will be collected weekly, will be saved as PDF files, and will be stored on the UFSCar institutional Google Drive. A researcher (R5) will be responsible for the data management. During the development of this research, the data will be restricted and for the exclusive use of registered researchers and participants. In addition, it will be available in folders saved in Google Drive. The confidential data of participants will not be available to the public.

#### Confidentiality {27}

The data will be stored on the institutional Google Drive of the Federal University of São Carlos (UFSCar). Only researchers from the UFSCar, who participate in this research, will have the authorization to access these stored documents. The metadata will be stored for the long term in the UFSCar research data repository.

#### Plans for collection, laboratory evaluation, and storage of biological specimens for genetic or molecular analysis in this trial/future use {33}

Not applicable to this research.

## Statistical methods

### Statistical methods for primary and secondary outcomes {20a}

An assessor blinded to the randomization and assessment processes will make the statistical inferences for the primary and secondary outcomes. The intention-to-treat principle will be adopted in the analyses. The normality of the data will be evaluated using the Kolmogorov-Smirnov test. Specific analyses will be used, depending on the distribution (normality and homogeneity) of the data. Parametric and nonparametric analyses will be used for normally and non-normally distributed data, respectively. These analyses will be performed using the SPSS v.17 statistical software (SPSS, Inc., IL, USA), considering a statistical power of 80% for all the tests, with a significance level of *p* < 0.05.

### Interim analyses {21b}

No provisional analyses will be performed. In cases of discontinuity, the intention-to-treat principle will be adopted.

### Methods for additional analyses (e.g., subgroup analyses) {20b}

No subgroups will be analyzed.

### Methods in analysis to handle protocol non-adherence and any statistical methods to handle missing data {20c}

In the case of discontinuity, missing data will be handled by adopting the intention-to-treat principle, in order to make statistical inferences.

### Plans to give access to the full protocol, participant-level data, and statistical code {31c}

Not applicable to this research.

### Oversight and monitoring

#### Composition of the coordinating center and trial steering committee {5d}

Not applicable to this research.

#### Composition of the data monitoring committee, its role, and reporting structure {21a}

Data monitoring committees are generally considered to better organize selected survey data. A researcher (R5) will be responsible for registering and organizing the data obtained in this research that will come from the documents and registration records: free and informed consent term (TCLE), screening, demographic data and clinical characteristics of the individuals participating in the research, Numerical Rating Scale, pressure pain threshold, conditioned pain modulation, temporal summation of pain, analgesic medication consumption during therapy, and global perceived effect. Metadata will be descriptive and administrative, derived from data collection, and made available in digital repositories. For metadata, the Dublin Core standard will be used.

#### Adverse event reporting and harms {22}

In the case of any harm or complication related to the treatments, this will be reported to the UFSCar Ethics Committee for Human Research. The same applies to possible ethical issues that may arise during the research. The guideline recommendations will be strictly followed.

#### Frequency and plans for auditing trial conduct {23}

Not applicable to this research.

#### Plans for communicating important protocol amendments to relevant parties (e.g., trial participants, ethical committees) {25}

This protocol was approved by the Human Research Ethics Committee (UFSCar) and registered in the Brazilian Registry of Clinical Trials - ReBEC (RBR-7vbw5gd). All amendments were described and submitted to the responsible centers, the university’s ethics committee, and ReBEC.

#### Dissemination plans {31a}

The results of this study will be published in a scientific journal. After that, the metadata will be deposited in the Institutional Repository of UFSCar (RI-UFSCar) and also disseminated to the local and national community through the press office of UFSCar, and in zenodo.org, for international Open Science access.

## Discussion

### Implications

The purpose of this study is to compare the TA and LA techniques, assessing which is most effective in reducing chronic nonspecific neck pain. Since the 1970s, the World Health Organization (WHO) has encouraged the use of TA [67], considered to be a low-cost therapy [24] that can provide clinical benefits relevant to neck pain [19, 36]. In contrast, there have been few studies of the use of LA for conditions involving neck pain [46]. Nonetheless, despite having been little studied for neck pain, it is an efficient analgesic and anti-inflammatory therapy that is totally painless, non-invasive, safe, and without adverse effects or complications. It can be used in locations where the insertion of needles may be complicated or risky [30, 33, 43] and is also a useful option for individuals with needle phobia.

### Strengths

Until the present moment, there are no studies in the literature that have effectively compared TA and LA in cases of chronic nonspecific neck pain. This research will be based on evaluating and comparing the changes in pain symptoms and the functional capacity of the neck, after the application of the treatments. The results obtained for the groups will be quantified and compared. Based on the findings, it will be possible to establish the therapeutic potential of each treatment, considering the outcomes of pain and cervical disability, making it possible to identify whether there is a difference between the techniques in the treatment of chronic nonspecific neck pain.

### Limitations

Some limitations include the inability to blind the researcher who will be responsible for applying the therapies, due to the nature of the interventions. To minimize a possible bias, this researcher will follow a script to standardize the treatments of all study groups. Individuals will be blinded to the true laser acupuncture and placebo intervention. However, due to the characteristics and differences of needle and laser, we will not be able to blind individuals between these therapies.

## Trial status

The protocol registration was approved on 6 June 2021. The initial recruitment date will be on 10 March 2021, and the approximate date of completion of the recruitment of participants will be up to the second half of 2022.

## Data Availability

Not applicable.

## Abbreviations

GPE: Global Perceived Effect Scale
CPM: Conditioned Pain Modulation
PPT: Pressure Pain Threshold
NRS: Numerical Rating Scale
TA: Traditional Acupuncture
LA: Laser Acupuncture
LLLT: Low-Level Laser Therapy
UFSCar: Federal University of São Carlos
ReBEC: Brazilian Registry of Clinical Trials
